# Sea cucumbers (Echinodermata, Holothuroidea) from the JR275 expedition to the eastern Weddell Sea, Antarctica

**DOI:** 10.3897/zookeys.1054.59584

**Published:** 2021-08-04

**Authors:** Melanie Mackenzie, P. Mark O’Loughlin, Huw Griffiths, Anton Van de Putte

**Affiliations:** 1 Sciences Department – Marine Invertebrates, Museums Victoria, GPO Box 666, Melbourne, Victoria 3001, Australia Museums Victoria Melbourne Australia; 2 British Antarctic Survey (BAS), High Cross Madingley Road, CB3 0ET, Cambridge, UK British Antarctic Survey Cambridge United Kingdom; 3 Royal Belgian Institute of Natural Sciences (RBINS), Rue Vautier 29, Brussels, Belgium Royal Belgian Institute of Natural Sciences Brussels Belgium

**Keywords:** Antarctic, benthic, biodiversity, dataset, holothuroid, Southern Ocean

## Abstract

Thirty-seven holothuroid species, including six potentially new, are reported from the eastern Weddell Sea in Antarctica. Information regarding sea cucumbers in this dataset is based on Agassiz Trawl (AGT) samples collected during the British Antarctic Survey cruise JR275 on the RRS *James Clark Ross* in the austral summer of 2012. Species presence by site and an appendix of holothuroid identifications with registrations are included as supplementary material. Species occurrence in the Weddell Sea is updated to include new holothuroids from this expedition.

## Introduction

The British Antarctic Survey (BAS) JR275 research cruise on the RRS *James Clark Ross* visited the Weddell Sea from February to March in 2012 as part of a core EvolHist (Evolutionary History of the Polar Regions) project. Prior to this expedition, the south-eastern Weddell Sea had been a relatively under sampled area on the Antarctic continental shelf, according to a gap analysis carried out by Griffiths et al. (2014). The eastern Weddell Sea is characterized by perennial sea ice cover and very large icebergs. The Filchner Trough is known to be an area responsible for generating the oxygen and nutrient-rich Antarctic Bottom Water (AABW), which helps drive oceanic circulation. By sampling benthic animals from the eastern Weddell Sea continental margin and slope and the deepened shelf basins of the Filchner Trough, the expedition aimed to collect specimens and associated data to investigate patterns of biodiversity and feed into biogeography and phylogeography studies of this important region of the Southern Ocean. Recording current biodiversity in the region is becoming increasingly urgent with the drastic decline in summer sea ice in the Weddell Sea over the last 5 years likely to have major implications for the marine ecosystem (Turner et al. 2020). Echinoids from JR275 were reported by Saucède et al. (2015) and the Asteroidea are included by Moreau et al. (2018).

This dataset reports holothuroid species occurrences and richness for individual Agassiz Trawls (AGTs) during the JR275 expedition and is provided for comparison with and updating of known lists of Weddell Sea and other Antarctic holothuroids. O’Loughlin et al. (2010) provided a comprehensive overview of Antarctic sea cucumber species, listing 187 species (including 51 still undescribed at the time of that publication) along with cryptic species and synonymies indicated by mtDNA sequence data. O’Loughlin et al. (2010) also reported 37 known species from shelf and slope depths in the Weddell Sea; here we add 11 new known species to this list for comparative depths (Table 5). The Weddell Sea is reported to be one of the most species-rich regions for holothuroids in Antarctica (O’Loughlin et al. 2010). Subsequent papers have continued to expand our knowledge of the previously undescribed Antarctic holothuroid fauna including papers by O’Loughlin and VandenSpiegel (2010) on apodids, O’Loughlin and Whitfield (2010) on psolids, O’Loughlin et al. (2013) on new holothuroids from Admiralty Bay, Davey and Whitfield (2013) on additional psolid fauna, O’Loughlin et al. (2014) on new Antarctic holothuroids and taxonomic reviews of some genera, Bohn and Hess (2014) on *Echinopsolus* and revisions within Psolidae and Cucumariidae, O’Loughlin et al. (2015a) on a new species of apodid from this JR275 expedition, and O’Loughlin et al. (2015b) on sea cucumbers of the Kerguelen Plateau. Gutt et al. (2014) also provided the original dataset for earlier voyages by German research vessel *Polarstern* to the Weddell Sea, including the holothuroid identifications and distributions published by Gutt (1990, 1991a, b) and subsequently referenced by O’Loughlin (2002), before further examination and revision by O’Loughlin et al. (2010). The dataset itself has since been updated by Piepenburg (2019). O’Loughlin et al. (2016) also gave an overview of Antarctic holothuroids collected during the historic *Discovery* expeditions. This current paper continues to build on our knowledge of Antarctic holothuroids.

This is a contribution to the SCAR (Scientific Committee on Antarctic Research) AntEco (State of the Antarctic Ecosystem) Programme.

## Design description

The dataset was published through the AntOBIS which is the Antarctic Marine Node of the international OBIS and GBIF, as a contribution to the EU-Lifewatch ERIC (https://www.lifewatch.eu/). Regarding the dataset, the Integrated Publishing Toolkit of the SCAR Antarctic Biodiversity Portal was used (http://ipt.biodiversity.aq/), following the Darwin Core event core. The dataset was uploaded in the AntOBIS (Antarctic Ocean Biogeographic Information System) database, and the taxonomy was matched against the Register of Antarctic Marine Species, using the Taxon Match tool (http://www.marinespecies.org/rams/aphia.php?p=match). The dataset meets the Darwin Core requirements and was designed around this event-core schema.

## Material and methods

**Figure 1.**
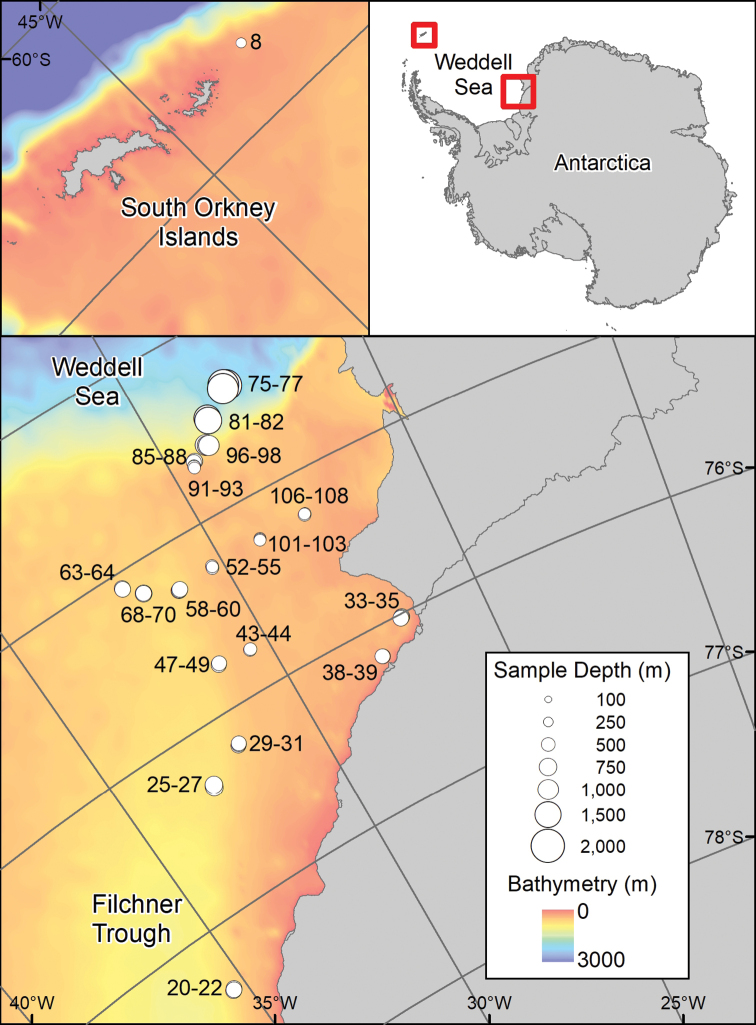
Sample locations for JR275 AGT holothuroid records.

Collecting equipment used on JR275 included an Agassiz trawl (AGT) and an Epibenthic Sledge (EBS) fitted with camera. This paper reports on the holothuroid specimens collected at 51 of the AGT sampling sites in the eastern Weddell Sea at depths of between ~400 and ~2,000 m, and a single test location at ~280 m depth off the South Orkney Islands (Fig. 1, Table 1). Weddell Sea deployments were mostly conducted along two transects, one running from south to north along the edge of the Filchner Trough and one running from west to east out of the Filchner Trough onto the shallower shelf. Over-deepened basins close to the Brunt Ice shelf were also sampled. At each site, three replicate AGT individual stations were taken and where the substrate was suitable a single EBS trawl was also conducted. EBS samples have not been examined for holothuroids at this stage and are not discussed further here, but this material is currently stored in the BAS collections in Cambridge, UK. The AGT used an inner mesh size of 1 cm, had a mouth width of 2 m, and was trawled at 1 knot for between 2 and 10 minutes depending on depth, substrate, and condition of animals in the initial catch. The deployment protocol was standardized and is outlined in full in the JR275 cruise report, available from the British Oceanographic Data Centre (https://www.bodc.ac.uk/resources/inventories/cruise_inventory/report/10598/).

**Table 1.** AGT sampling stations where holothuroids were collected on JR275.

Deployment number	Start latitude	End latitude	Start longitude	End longitude	Minimum depth (m)	Maximum depth (m)	Date
8*	−60.68	−60.68	−44.01	−44.01	279.04	281.57	11/2/2012
20	−77.36	−77.36	−35.37	−35.36	654.34	654.35	19/2/2012
21	−77.35	−77.35	−35.35	−35.34	648.18	652.80	19/2/2012
22	−77.35	−77.35	−35.33	−35.32	650.78	654.20	19/2/2012
25	−76.33	−76.33	−32.90	−32.90	778.81	781.73	20/2/2012
26	−76.32	−76.32	−32.88	−32.88	780.30	789.24	20/2/2012
27	−76.32	−76.31	−32.87	−32.87	779.51	781.36	20/2/2012
29	−76.20	−76.20	−31.86	−31.86	575.95	578.97	20/2/2012
30	−76.20	−76.19	−31.84	−31.84	575.99	578.94	20/2/2012
31	−76.19	−76.19	−31.82	−31.82	564.11	573.00	20/2/2012
33	−76.02	−76.02	−27.00	−26.99	605.21	610.00	21/2/2012
34	−76.02	−76.02	−26.98	−26.97	608.00	613.00	21/2/2012
35	−76.02	−76.02	−26.96	−26.96	607.00	613.01	21/2/2012
38	−76.17	−76.17	−27.80	−27.80	544.89	561.00	21/2/2012
39	−76.17	−76.17	−27.80	−27.80	549.28	555.26	21/2/2012
43	−75.76	−75.76	−30.45	−30.45	427.94	430.00	22/2/2012
44	−75.77	−75.77	−30.46	−30.46	429.39	436.80	22/2/2012
47	−75.74	−75.74	−31.24	−31.24	578.94	584.88	22/2/2012
48	−75.75	−75.75	−31.25	−31.25	584.83	590.75	22/2/2012
49	−75.75	−75.75	−31.26	−31.27	583.36	584.94	22/2/2012
52	−75.24	−75.24	−30.25	−30.25	418.73	419.21	23/2/2012
53	−75.25	−75.25	−30.25	−30.25	417.39	417.78	23/2/2012
54	−75.25	−75.25	−30.26	−30.26	418.70	419.11	23/2/2012
55	−75.26	−75.26	−30.26	−30.27	418.38	418.61	23/2/2012
58	−75.26	−75.26	−31.13	−31.13	604.29	607.13	23/2/2012
59	−75.27	−75.27	−31.14	−31.15	607.10	610.24	23/2/2012
60	−75.27	−75.27	−31.16	−31.17	614.30	616.52	23/2/2012
63	−75.09	−75.09	−32.22	−32.22	609.48	612.28	24/2/2012
64	−75.09	−75.09	−32.22	−32.22	610.62	611.83	24/2/2012
68	−75.18	−75.18	−31.87	−31.87	655.78	676.11	24/2/2012
69	−75.18	−75.18	−31.87	−31.87	654.87	657.46	24/2/2012
70	−75.17	−75.18	−31.87	−31.87	654.65	691.31	24/2/2012
75	−74.37	−74.37	−28.11	−28.10	2052.26	2053.91	26/2/2012
76	−74.38	−74.38	−28.07	−28.06	2056.14	2058.19	26/2/2012
77	−74.39	−74.39	−28.16	−28.15	2006.54	2011.16	26/2/2012
81	−74.51	−74.51	−28.75	−28.74	1558.28	1570.08	28/2/2012
82	−74.50	−74.49	−28.74	−28.74	1580.27	1595.46	28/2/2012
85	−74.67	−74.68	−29.42	−29.43	586.74	604.49	29/2/2012
86	−74.68	−74.68	−29.45	−29.45	573.42	580.99	29/2/2012
88	−74.67	−74.67	−29.43	−29.43	592.71	602.27	29/2/2012
91	−74.71	−74.71	−29.51	−29.51	401.67	410.00	29/2/2012
92	−74.70	−74.70	−29.50	−29.50	427.17	428.55	29/2/2012
93	−74.70	−74.70	−29.50	−29.50	439.76	450.09	29/2/2012
96	−74.63	−74.63	−29.05	−29.04	1018.91	1028.48	1/3/2012
97	−74.63	−74.63	−29.02	−29.02	985.75	1010.63	1/3/2012
98	−74.64	−74.64	−29.00	−28.99	941.94	971.14	1/3/2012
101	−75.24	−75.24	−29.00	−29.01	391.66	398.30	4/3/2012
102	−75.25	−75.25	−29.02	−29.02	392.77	396.83	4/3/2012
103	−75.25	−75.25	−29.03	−29.03	390.17	392.20	4/3/2012
106	−75.24	−75.24	−27.85	−27.85	413.67	415.71	4/3/2012
107	−75.24	−75.24	−27.86	−27.87	414.23	415.15	4/3/2012
108	−75.24	−75.24	−27.88	−27.88	417.56	424.41	4/3/2012

* Note: Site 8 is in the South Orkneys, all other sites are eastern Weddell Sea.

Live or early preservation images of specimens were taken on board the RRS *James Clark Ross* by Camille Moreau (Institut Universitaire Europeen de la Mer, Brest, France) with assistance from Melanie Mackenzie (Museums Victoria), James Rudd (BAS), and Douglas Hamilton (University of East Anglia) using a digital SLR Nikon D3X with 60 mm lens and flash rigging and Nikon D700 with 60 mm lens.

Preliminary identification of holothuroid material was carried out during the cruise by Melanie Mackenzie. DNA tissue samples (chiefly tentacle or tube foot sub-samples) were taken by Melanie Mackenzie with assistance from Stuart MacMillan (BAS) for BOLD barcoding. A total of 190 specimens from 50 AGT stations (~15% of all specimens) were sampled during the cruise. DNA extractions of some Elpidiidae holothuroid specimens were conducted onboard by Jennifer Jackson (BAS) with assistance from Melanie Mackenzie using QIAGEN DNeasy Blood & Tissue molecular kit. Tissues samples of *Peniagone* specimens were also sent to Pamela Brannock at Auburn University and Rollins College in USA and a sample of *Protelpidia* was sent to Akito Ogawa at the National Museum of Nature and Science in Japan for further genetic analysis. Holothuroid specimens were preserved in 96% undenatured and pre-cooled (at −20 °C) ethanol and subsequently transferred to −20 °C freezer for a minimum of 48 hours with rotation of containers to ensure full preservation of material. Material was sent on to Melanie Mackenzie and Mark O’Loughlin at Museums Victoria, in Australia, for further examination and determination using stereo and compound microscopes. New species of Apodida (as Synaptida) specimens from this collection were described in O’Loughlin et al. (2015a) and are reported in Table 5 and Suppl. material 1: Table S1 alongside determinations of the remaining sea cucumber samples from AGT trawls. Specimens were identified to species level where possible, and to higher taxonomic levels where species-level determinations could not be made. Determinations here are based on morpho-taxonomic methods. Future genetic and morphological work may lead to additions or changes.

### Abbreviations

**AntaBIS** Antarctic Biodiversity Information System;

**AntEco** State of the Antarctic Ecosystem;

**AntOBIS** Antarctic Thematic Node of the Ocean Biogeographic Information System;

**BAS** British Antarctic Survey;

**EvolHist** Evolutionary History of the Polar Regions (a BAS core project);

**NHMUK**British Museum of Natural History (registration number prefix NHMUK);

**NMV** Museums Victoria, Australia, used with registration number prefix F.;

**SCAR** The Scientific Committee on Antarctic Research.

## Taxonomic coverage

**Remarks.**Miller et al. (2017) assessed and revised the phylogeny of extant Holothuroidea. Apodida was highlighted as a sister to the rest of Holothuroidea, the previously paraphyletic Aspidochirotida was split into Molpadida, Dendrochirotida, and Elasipodida (in part) and a new order, Holothuriida, was erected. For this paper, we follow the taxonomic groups put forward by Miller et al. (2017) and currently accepted in the World Register of Marine Species database (WoRMS 2020).

**General taxonomic coverage description.** The present dataset focuses on the class Holothuroidea (Echinodermata). Of the seven orders currently sitting within class Holothuroidea, six are represented, with only Molpadiida not being collected in the AGT catches on this voyage. This dataset looks at over 1200 specimens belonging to 10 families, and at least 23 genera and 31 species.

**Phylum**: Echinodermata

**Class**: Holothuroidea

**Orders**: Apodida, Dendrochirotida, Elasipodida, Holothuriida, Molpadida, Persiculida, Synallactida

**Families**: Chiridotidae, Cucumariidae, Paracucumidae, Psolidae, Elpidiidae, Laetmogonidae, Mesothuriidae, Molpadiodemidae, Pseudostichopodidae, Synallactidae

**Genera**: *Paradota*, *Sigmodota*, *Taeniogyrus*, *Echinopsolus*, *Heterocucumis*, *Parathyonidium*, *Pentactella*, *Psolicrux*, *Staurocucumis*, *Trachythyone*, *Crucella*, *Paracucumis*, *Psolidium*, *Psolus*, *Elpidia*, *Peniagone*, *Protelpidia*, *Rhipidothuria*, *Laetmogone*, *Mesothuria*, *Molpadiodemas*, *Pseudostichopus*, *Bathyplotes*

**Species**: *Paradotaweddellensis. Sigmodotamagdarogera*, *Sigmodotamagnibacula*, *Taeniogyrusbamberi*, *Echinopsolusacutus*, *Echinopsolusmollis*, *Heterocucumissteineni*, *Parathyonidiumincertum*, *Psolicruxiuvenilesi*, *Staurocucumisliouvillei*, *Trachythyonebouvetensis*, *Trachythyonecynthiae*, *Crucellahystrix*, *Paracucumisturricata*, *Psolidiumgaini*, *Psolidiumtenue*, *Psolidiumwhittakeri*, *Psolusdubiosus*, *Psoluslockhartae*, *Elpidiaglacialis*, *Peniagoneincerta*, *Peniagonevignoni*, *Protelpidiamurrayi*, *Rhipidothuriaracovitzai*, *Laetmogonewyvillethomsoni*, *Mesothuriabifurcata*, *Molpadiodemascrinitus*, *Pseudostichopusspiculiferus*, *Pseudostichopusperipatus* complex, *Bathyplotesbongraini*, *Bathyplotesmoseleyi*.

**Other**: The following specimens with suffix sp. 1 belong to none of the known species listed in the dataset and will likely be described as new species after further morphological and genetic analyses: Cucumariidae sp. 1 (sp Mov 7265), *Echinopsolus* sp. 1 (sp Mov 7266), *Pentactella* sp. 1 (sp Mov 7267), *Staurocucumis* sp. 1 (sp Mov 7268), *Psolus* sp. 1 (sp Mov 7269), and *Peniagone* sp. 1 (sp Mov 7270).

In Tables 2–4, specimens identified as cf. species or only identified to a higher taxonomic level, i.e. species indeterminate (sp. indet.), genus indeterminate (gen. indet.), and family indeterminate (fam. indet.), are recorded on separate rows.

**Table 2.** Presence-only matrix of sea cucumber species in AGT trawls on JR275, Stations 8 to 49 (only stations with holothuroids included).

Order	Family	Genus	Species	JR275 Station Number / Site																			
				8	20	21	22	25	26	27	29	30	31	33	34	35	38	39	43	44	47	48	49
** APODIDA **	** Chiridotidae **	* Paradota *	* weddellensis *																				
		* Sigmodota *	* magdarogera *																				
		* Sigmodota *	* magnibacula *														X	X					
		* Taeniogyrus *	* bamberi *																				
** DENDROCHIROTIDA **	** Cucumariidae **	Cucumariidae	sp.1															X					
		* Echinopsolus *	* acutus *														X	X					
		* Echinopsolus *	* mollis *															X					
		* Echinopsolus *	cf. mollis														X						
		* Echinopsolus *	sp.1																				
		* Echinopsolus *	sp. indet.																				
		* Heterocucumis *	steineni																				
		* Parathyonidium *	* incertum *	X																			
		* Pentactella *	sp.1											X									
		* Psolicrux *	* iuvenilesi *														X	X					
		* Psolicrux *	sp. indet.																				
		* Staurocucumis *	* liouvillei *	X							X	X	X	X	X		X	X	X				X
		* Staurocucumis *	sp. 1														X	X				X	
		* Trachythyone *	* bouvetensis *			X		X	X	X	X	X				X						X	
		* Trachythyone *	* cynthiae *												X		X	X					
		Cucumariidae	gen. indet.																				
	** Paracucumidae **	* Crucella *	* hystrix *														X						
		* Paracucumis *	* turricata *			X														X	X		
	** Psolidae **	* Psolidium *	* gaini *																				
		* Psolidium *	* tenue *			X						X						X					
		* Psolidium *	* whittakeri *																				
		* Psolidium *	sp. indet.																				
		* Psolus *	* dubiosus *								X	X	X				X	X				X	X
		* Psolus *	cf. dubiosus																				
		* Psolus *	* lockhartae *																				
		* Psolus *	sp. 1																				
		Psolidae	gen. indet.																				
		Dendrochirotida	fam. indet.																				
** ELASIPODIDA **	** Elpidiidae **	* Elpidia *	* glacialis *		X	X					X	X	X										X
		* Peniagone *	sp. 1																				
		* Peniagone *	* incerta *																				
** ELASIPODIDA **	** Elpidiidae **	* Peniagone *	cf. incerta																				
		* Peniagone *	* vignoni *			X	X	X	X	X	X	X	X			X	X	X		X	X		X
		* Protelpidia *	* murrayi *										X								X		
		* Rhipidothuria *	* racovitzai *		X	X	X							X	X	X					X	X	
		Elpidiidae	gen. indet.																				
	** Laetmogonidae **	* Laetmogone *	* wyvillethomsoni *					X	X														
** HOLOTHURIIDA **	** Mesothuriidae **	* Mesothuria *	* bifurcata *		X			X	X	X	X	X	X					X		X	X	X	X
** PERSICULIDA **	** Molpadiodemidae **	* Molpadiodemas *	* crinitus *										X										
	** Pseudostichopodidiae **	* Pseudostichopus *	* spiculiferus *	X														X					
		* Pseudostichopus *	*peripatus* complex																				
** SYNALLACTIDA **	** Synallactidae **	* Bathyplotes *	* bongraini *																				
		* Bathyplotes *	* moseleyi *					X		X													
		* Bathyplotes *	sp. indet.					X	X														
		Synallactidae	gen. indet.																				

**Table 3.** Presence-only matrix of sea cucumber species in AGT trawls on JR275, Stations 52 to 88 (only stations with holothuroids included).

Order	Family	Genus	Species	JR275 Station Number / Site																			
				52	53	54	55	58	59	60	63	64	68	69	70	75	76	77	81	82	85	86	88
** APODIDA **	** Chiridotidae **	* Paradota *	* weddellensis *													X		X					
		* Sigmodota *	* magdarogera *						X			X											
		* Sigmodota *	* magnibacula *																				
		* Taeniogyrus *	* bamberi *							X													
** DENDROCHIROTIDA **	** Cucumariidae **	Cucumariidae	sp. 1					X	X	X													
		* Echinopsolus *	* acutus *																		X		
		* Echinopsolus *	* mollis *																				
		* Echinopsolus *	cf. mollis																				
		* Echinopsolus *	sp. 1																		X		
		* Echinopsolus *	sp. indet.																				X
		* Heterocucumis *	* steineni *																		X		X
		* Parathyonidium *	* incertum *																				
		* Pentactella *	sp. 1																				
		* Psolicrux *	* iuvenilesi *							X											X	X	
		* Psolicrux *	sp. indet.																				
		* Staurocucumis *	* liouvillei *	X				X		X	X										X	X	X
		* Staurocucumis *	sp. 1																				
		* Trachythyone *	* bouvetensis *							X													
		* Trachythyone *	* cynthiae *																		X		
		Cucumariidae	gen. indet.	X																		X	
	** Paracucumidae **	* Crucella *	* hystrix *					X															
		* Paracucumis *	* turricata *																				
	** Psolidae **	* Psolidium *	* gaini *																		X		
		* Psolidium *	* tenue *			X		X					X										
		* Psolidium *	* whittakeri *																		X		
		* Psolidium *	sp. indet.																				
		* Psolus *	* dubiosus *						X												X		
		* Psolus *	cf. dubiosus								X												
		* Psolus *	* lockhartae *													X	X	X					
		* Psolus *	sp. 1													X					X		
		Psolidae	gen. indet.																			X	X
		Dendrochirotida	fam. indet.	X																			X
** ELASIPODIDA **	** Elpidiidae **	* Elpidia *	* glacialis *								X		X										
		* Peniagone *	sp. 1																	X			
		* Peniagone *	* incerta *													X	X	X					
		* Peniagone *	cf. incerta													X		X	X				
		* Peniagone *	* vignoni *	X	X	X	X	X					X	X									
		* Protelpidia *	* murrayi *								X				X								
		* Rhipidothuria *	* racovitzai *																				
		Elpidiidae	gen. indet.	X																			
	** Laetmogonidae **	* Laetmogone *	* wyvillethomsoni *																				
** HOLOTHURIIDA **	** Mesothuriidae **	* Mesothuria *	* bifurcata *				X	X	X	X	X			X									
** PERSICULIDA **	** Molpadiodemidae **	* Molpadiodemas *	* crinitus *	X																			
	** Pseudostichopodidiae **	* Pseudostichopus *	* spiculiferus *										X										
		* Pseudostichopus *	*peripatus* complex													X		X					
** PERSICULIDA **	** Synallactidae **	* Bathyplotes *	* bongraini *																				
		* Bathyplotes *	* moseleyi *					X	X										X				
		* Bathyplotes *	sp. indet.							X													
		Synallactidae	gen. indet.						X														

**Table 4.** Presence-only matrix of sea cucumber species in AGT trawls on JR275, Stations 91 to 108 (only stations with holothuroids included).

Order	Family	Genus	Species	91	92	93	96	97*	98	101	102	103	106	107	108
** APODIDA **	** Chiridotidae **	* Paradota *	* weddellensis *												
		* Sigmodota *	* magdarogera *												
		* Sigmodota *	* magnibacula *												
		* Taeniogyrus *	* bamberi *			X									
** DENDROCHIROTIDA **	** Cucumariidae **	Cucumariidae	sp. 1												
		* Echinopsolus *	* acutus *												
		* Echinopsolus *	* mollis *												
		* Echinopsolus *	cf. mollis												
		* Echinopsolus *	sp. 1												
		* Echinopsolus *	sp. indet.												
		* Heterocucumis *	* steineni *												
		* Parathyonidium *	* incertum *												
		* Pentactella *	sp. 1												
		* Psolicrux *	* iuvenilesi *												
		* Psolicrux *	sp. indet.	X											
		* Staurocucumis *	* liouvillei *				X	X	X				X		X
		* Staurocucumis *	sp. 1												
		* Trachythyone *	* bouvetensis *												
		* Trachythyone *	* cynthiae *												
		Cucumariidae	gen. indet.	X	X										
	** Paracucumidae **	* Crucella *	* hystrix *												
		* Paracucumis *	* turricata *									X			
	** Psolidae **	* Psolidium *	* gaini *												
		* Psolidium *	* tenue *					X	X						
		* Psolidium *	* whittakeri *				X								
		* Psolidium *	sp. indet.		X										
		* Psolus *	* dubiosus *				X				X				
		* Psolus *	cf. dubiosus						X						
		* Psolus *	* lockhartae *												
		* Psolus *	sp. 1												
		Psolidae	gen. indet.	X	X										
		Dendrochirotida	fam. indet.	X	X	X				X					
** ELASIPODIDA **	** Elpidiidae **	* Elpidia *	* glacialis *												
		* Peniagone *	sp. 1												
		* Peniagone *	* incerta *												
		* Peniagone *	cf. incerta												
		* Peniagone *	* vignoni *								X	X	X	X	X
		* Protelpidia *	* murrayi *												
		* Rhipidothuria *	* racovitzai *												
		Elpidiidae	gen. indet.							X					
	** Laetmogonidae **	* Laetmogone *	* wyvillethomsoni *												
** HOLOTHURIIDA **	** Mesothuriidae **	* Mesothuria *	* bifurcata *								X		X		X
** PERSICULIDA **	** Molpadiodemidae **	* Molpadiodemas *	* crinitus *												
	** Pseudostichopodidiae **	* Pseudostichopus *	* spiculiferus *										X		
		* Pseudostichopus *	*peripatus* complex												
** SYNALLACTIDA **	** Synallactidae **	* Bathyplotes *	* bongraini *								X				
		* Bathyplotes *	* moseleyi *				X	X	X						
		* Bathyplotes *	sp. indet.												
		Synallactidae	gen. indet.												

*Holothuroidea indet. (previously Aspidochirotida) also present at AGT 97.

## Spatial coverage

**General spatial coverage**: East Weddell Sea, Antarctica.

**Coordinates**: 60.68°S and 77.36°S; 44.01°W and 26.78°W.

**Temporal coverage**: February 12, 2012–March 4, 2012.

## Natural collections description

**Initial collection identifier**: British Antarctic Survey.

**Collection name**: EvolHist JR275 Weddell Sea Holothuroids.

**Final Lodgment Institutions**: British Antarctic Survey (BAS), Natural History Museum UK (NHMUK), Museums Victoria (NMV). Location and Registration Numbers as per Suppl. material 1: Table S1.

**Collection identifier**: O’Loughlin and Mackenzie.

**Specimen preservation method**: Ethanol (original fixative 95%).

**Remarks.** A diverse holothuroid assemblage was collected, with over 1,200 holothuroids (~13.5 kg) from the Agassiz trawls alone, making these echinoderms one of the most abundant groups collected during the voyage and reinforcing previous records of high holothuroid abundance and diversity in this area. Holothuroids were found in 51 of the possible 55 AGTs, including in the iceberg scoured ‘graveyards’ of events 91 to 93.

**Table 5.** Holothuroid species reported from the Weddell Sea to 1180 m (following O’Loughlin et al. 2010).

** APODIDA **
** Chiridotidae **
*Paradotaweddellensis* Gutt, 1990
*Sigmodotamagdarogera* O’Loughlin in O’Loughlin et al. 2015
*Sigmodotamagnibacula* (Massin & Hétérier, 2004)
*Taeniogyrusbamberi* O’Loughlin in O’Loughlin et al. 2015
*Taeniogyruscontortus* (Ludwig, 1875)
** DENDROCHIROTIDA **
** Cucumariidae **
*Cucambapsolidiformis* (Vaney, 1908)
“*Cucumariageorgiana* (Lampert, 1886) group” (by Gutt 1990)
*Echinopsolusacanthocola* Gutt, 1990
*Echinopsolusacutus* (Massin, 1992)
*Echinopsoluscharcoti* (Vaney, 1906)
*Echinopsolusmollis* (Ludwig & Heding, 1935)
*Echinopsolusparvipes* Massin, 1992
*Echinopsolussplendidus* (Gutt, 1990)
*Heterocucumisdenticulata* (Ekman, 1927)
*Heterocucumissteineni* (Ludwig, 1898)
*Parathyonidiumincertum* Heding in Heding and Panning 1954
*Psolicruxcoatsi* (Vaney, 1908)
*Psolicruxiuvenilesi* O’Loughlin & Manjón-Cabeza, 2009
*Staurocucumisliouvillei* (Vaney, 1914)
*Staurocucumisturqueti* (Vaney, 1906)
*Trachythyonebouvetensis* (Ludwig & Heding, 1935)
*Trachthyonecynthiae* O’Loughlin, 2009
*Trachythyonemaxima* Massin, 1992
*Trachythyoneparva* (Ludwig, 1875)
** Paracucumidae **
*Crucellahystrix* Gutt, 1990
*Crucellascotiae* (Vaney, 1906)
*Paracucumisturricata* (Vaney, 1906)
** Psolidae **
*Psolidiumgaini* Vaney, 1914
*Psolidiumpawsoni* O’Loughlin & Ahearn, 2008
*Psolidiumtenue* Mortensen, 1925
*Psolidiumwhittakeri* O’Loughlin & Ahearn, 2008
*Psolusantarcticus* (Philippi, 1857)
*Psolusdubiosus* Ludwig & Heding, 1935
Psoluscf.lockhartae (O’Loughlin & Whitfield, 2010)*
** ELASIPODIDA **
** Elpidiidae **
*Elpidiaglacialis* Théel, 1876
*Peniagoneincerta* (Théel, 1882)
*Peniagonevignoni* Hérouard, 1901
*Protelpidiamurrayi* (Théel, 1879)
*Rhipidothuriaracovitzai* Hérouard, 1901
** Laetmogonidae **
*Laetmogonewyvillethomsoni* Théel, 1879
** PERSICULIDA **
** Mesothuriidae **
*Mesothuriabifurcata* Hérouard, 1901
** MOLPADIDA **
** Molpadiidae **
*Molpadiamusculus* Risso, 1826
** PERSICULIDA **
** Molpadiodemidae **
*Molpadiodemascrinitus* O’Loughlin & Ahearn, 2005
** Pseudostichopodidae **
*Pseudostichopusspiculiferus* (O’Loughlin, 2002)
*Pseudostichopusperipatus* (Sluiter, 1901) complex
** SYNALLACTIDA **
** Synallactidae **
*Bathyplotesbongraini* Vaney, 1914
*Bathyplotesgourdoni* (Vaney, 1914)
*Bathyplotesmoseleyi* (Théel, 1886)

*Note: Psoluscf.lockhartae was found at shallower comparative depths and is noted in the table above.

**Plus, potential new species at comparative depths**:

*Cucumariidae* sp. 1, *Echinopsolus* sp. 1, *Pentactella* sp. 1, *Staurocucumis* sp. 1, *Psolus* sp. 1 and *Peniagone* sp. 1.

**Plus, one known species at greater depth**: (~2000 m) *Psoluslockhartae* (O’Loughlin & Whitfield, 2010).

## Datasets

**Dataset description**: Biodiversity.aq – Integrated Publishing Toolkit (IPT version 2.4.0).

**Object name**: Sea cucumbers (Echinodermata, Holothuroidea) from the JR275 expedition to the Eastern Weddell Sea, Antarctica – Data.

**Character encoding**: UTF-8.

**Format name**: Darwin Core Archive format.

**Format version**: 1.0.

**Distribution**: https://ipt.biodiversity.aq/manage/resource.do?r=bas_jr275_holothuroidea. Mackenzie M, O’Loughlin PM, Griffiths H, Van de Putte AP, Van de Putte A (2021) Sea cucumbers (Echinodermata, Holothuroidea) from the JR275 expedition to the Eastern Weddell Sea, Antarctica – Data. SCAR – AntOBIS. Occurrence dataset https://hes32-ctp.trendmicro.com:443/wis/clicktime/v1/query?url=https%3a%2f%2fdoi.org%2f10.15468%2f64c2ha&umid=2f96c605-47bc-4ec1-9c10-8ab8afdd2922&auth=89a422ce48cf9afc268cabe806cc53ea452e36bd-028570941b7b4372c9f0db6ba972ca8c781fba68 accessed via GBIF.org on 2021-05-23. https://www.gbif.org/dataset/fcc25f03-8437-4ea3-859e-67866de5cb80

**Publication date of data**: [pending]

**Language**: English

**Metadata language**: English

**Date of metadata creation**: Last modified Oct 7, 2020

**Hierarchy level**: Dataset

## Supplementary materials

Supplementary material 1 Table S1 Data type table (xlsx. file) Explanation note Full list of identifications with registration numbers and institutions. https://binary.pensoft.net/file/574357This dataset is made available under the Open Database License (http://opendatacommons.org/licenses/odbl/1.0/). The Open Database License (ODbL) is a license agreement intended to allow users to freely share, modify, and use this Dataset while maintaining this same freedom for others, provided that the original source and author(s) are credited.Melanie Mackenzie, P. Mark O’Loughlin, Huw Griffiths, Anton Van de Putte
